# Effectiveness of platelet-rich plasma therapy in promoting wound healing and shoulder function recovery after shoulder surgery

**DOI:** 10.1038/s41598-026-44844-3

**Published:** 2026-03-27

**Authors:** Zheng Hu, Licheng Wei, Weiguo Hu, Xing Li, Zihao Ren, Guangqing Cai, Chang Lan, Yilin Zhu, Hepeng Yang

**Affiliations:** 1 Department of Orthopedics, Changsha Hospital of Traditional Chinese Medicine, Changsha Eighth Hospital, 22 Xingsha Avenue, Changsha, 410199 Hunan Province China; 2 Department of Orthopedics, The Fourth Hospital of Changsha, Changsha, 410000 Hunan Province China

**Keywords:** PRP therapy, Shoulder surgery, Wound healing, Shoulder joint function recovery, Postoperative pain management, Diseases, Health care, Medical research, Risk factors

## Abstract

This retrospective propensity score–matched cohort study examined whether Platelet-Rich Plasma (PRP) use was associated with improved wound healing and functional recovery after shoulder surgery. We analyzed records from Changsha Hospital of Traditional Chinese Medicine (January 2018–December 2023) and included 170 patients undergoing rotator cuff repair (n = 100), shoulder replacement (n = 40), or subacromial decompression (n = 30). Within each surgery type, patients receiving PRP (treatment group [TG]) were matched 1:1 to patients not receiving PRP (control group [CG]) using propensity scores derived from age, sex, comorbidities, and baseline pain. Outcomes included wound healing time, postoperative pain (Visual Analog Scale [VAS]), shoulder function (Constant–Murley score), complications, length of hospital stay, and patient satisfaction. In matched analyses, PRP use was associated with shorter wound healing time and improved postoperative pain and functional outcomes across procedures, with the largest and most robust differences observed in the rotator cuff repair cohort. PRP was also associated with a shorter hospitalization duration in all three surgery cohorts. Complication rates were lower in the rotator cuff repair cohort, while no clear between-group differences were observed in the shoulder replacement or subacromial decompression cohorts. Overall patient satisfaction was higher in the PRP groups. Given the retrospective design, potential residual confounding, and smaller sample sizes in non–rotator cuff cohorts, these findings should be interpreted cautiously and warrant confirmation in prospective, surgery-specific studies using standardized PRP protocols.

**Supplementary Information:**

The online version contains supplementary material available at 10.1038/s41598-026-44844-3.

## Introduction

In recent years, Platelet-Rich Plasma (PRP) therapy, as a novel approach to biological treatment, has been widely concerned in the fields of orthopedics and sports medicine^1^. PRP is a kind of plasma extracted and concentrated from patients’ own blood by centrifugation, which is rich in many platelets and growth factors, such as transforming growth factor-β (TGF-β), vascular endothelial growth factor (VEGF) and Platelet-Derived Growth Factor (PDGF). These growth factors play a critical role in tissue repair and regeneration by promoting cell proliferation, angiogenesis, and collagen synthesis, thereby accelerating wound healing and tissue restoration^2–5^. At present, PRP therapy is employed in management of various soft tissue injuries, including the repair of tendons, ligaments, and muscles^6–8^.

The human shoulder is made up three bones, which is often affected by sports injury^9^ or degenerative diseases^10^. As a common surgical treatment, shoulder surgery is widely used to treat a range of injuries and illnesses affecting the shoulders, such as rotator cuff tear, shoulder inflammation, subacromial impingement syndrome and shoulder instability^11,12^. Despite the advancements in modern surgical technology that have considerably raised surgical success rates and postoperative patient quality of life, poor wound healing and slow functional recovery remain major challenges in the rehabilitation process^11^. To support the postoperative recovery effect, the application of PRP therapy in orthopedic surgery has gradually attracted attention in recent years^13,14^.

PRP therapy has the potential to accelerate tissue healing and is associated with reduced inflammatory response^15^. In the recovery process after shoulder surgery, PRP therapy has shown potential in promoting wound healing and the recovery of shoulder function^16^. Although PRP therapy has theoretical advantages, its effectiveness in practical application after shoulder surgery remains controversial. It is imperative that further study be done to support the true benefits of PRP treatment for shoulder surgery.

This study adopts a single-center, retrospective design and analyzes the patient data from Changsha Hospital of Traditional Chinese Medicine between 2018 and 2023 for shoulder surgery patients who also had PRP treatment. Using PS matching strategy, the CG patients with similar clinical characteristics were selected and matched at a ratio of 1:1. The aim was to assess scientifically the effectiveness of PRP therapy in promoting wound healing and shoulder joint function recovery after shoulder surgery, providing a rationale for the therapeutic use of PRP in shoulder surgery.

## Materials and methods

### General information

**Fig. 1:**
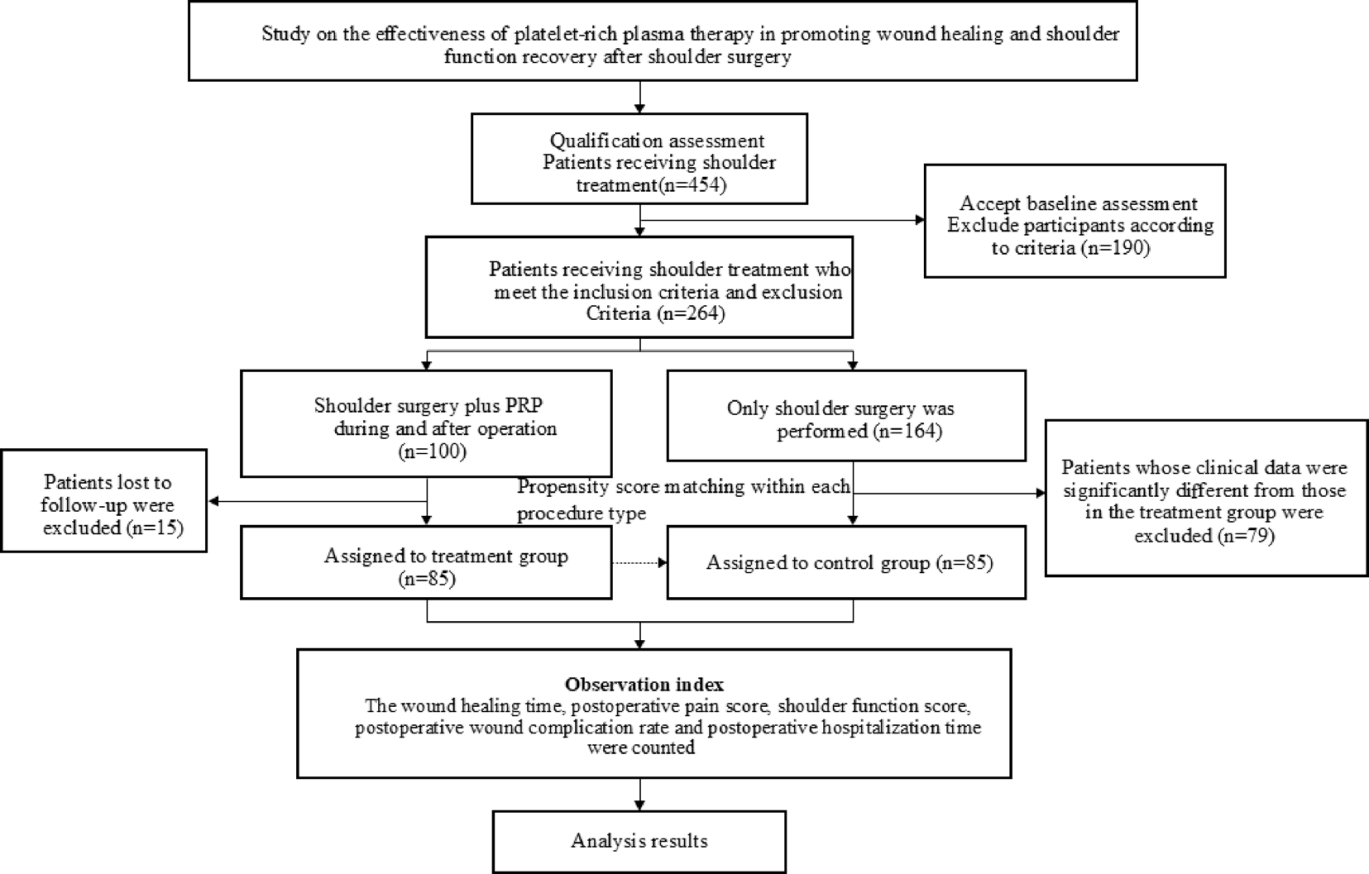
Flow chart of research queue screening.

This retrospective cohort study analyzed data from the electronic Hospital Information System (HIS) of Changsha Hospital of Traditional Chinese Medicine between January 2018 and December 2023. Eligible patients undergoing rotator cuff repair, shoulder replacement, or subacromial decompression were identified. Patients who received PRP per protocol comprised the treatment group (TG), and those who did not receive PRP comprised the control group (CG). Within each procedure type, TG and CG were matched 1:1 using propensity scores (nearest-neighbor matching; caliper = 0.2) based on age, sex, comorbidities, shoulder pathology, and baseline Visual Analog Scale (VAS) score, yielding matched cohorts of 50 TG/50 CG for rotator cuff repair, 20 TG/20 CG for shoulder replacement, and 15 TG/15 CG for subacromial decompression (total N = 170). The study was approved by the Ethics Committee of Changsha Hospital of Traditional Chinese Medicine , and the requirement for informed consent was waived due to the retrospective study design. All methods were performed in accordance with the relevant guidelines and regulations, including institutional policies for human research, data protection, and patient confidentiality. All data extracted from the Hospital Information System were de-identified prior to analysis, and the study complied with the Declaration of Helsinki (as revised). Figure 1 presents the study cohort selection flow diagram.

Inclusion criteria:

1. Patients who are adults and at least eighteen.
2. Patients who opted for surgical treatment after ineffective regular conservative treatment for more than 3 months.
3. Types of surgery: rotator cuff repair, shoulder replacement, and subacromial decompression.
4. No contraindications such as shoulder joint infection or severe joint contracture.
5. Complete clinical data, including operation records, postoperative follow-up records, imaging examination results, and laboratory examination data.

Exclusion criteria:

1. Serious systemic diseases, such as serious cardiovascular diseases, renal insufficiency, or liver dysfunction.
2. Patients with infectious diseases, including acute or chronic infection before or after operation.
3. Patients with a history of allergies to PRP therapy or its components.
4. Patients with immune system diseases, such as autoimmune diseases or immunodeficiency diseases.
5. Patients with malignant tumors, a history of malignant tumors, recent malignancy, or those currently undergoing treatment for malignancy.
6. Pregnant and lactating women.
7. Patients receiving drug treatments (such as long-term use of steroids or anticoagulants) that may affect wound healing or shoulder joint function recovery;
8. Patients with a history of mental disorders.

### Sample size considerations

Because this was a retrospective single-center matched cohort, no a priori sample size calculation was possible. We therefore used a minimum detectable difference approach to characterize statistical precision for the principal efficacy endpoint, wound-healing time, in the largest matched surgical subgroup, the rotator cuff repair cohort. Under a two-sided α of 0.05, 80% power, 50 matched patients per group, and a pooled standard deviation of approximately 0.71 days, the minimum detectable between-group difference was approximately 0.40 days. The observed between-group difference in wound-healing time in this subgroup was 1.4 days (95% CI, 1.1 to 1.7), which was substantially larger than this threshold. A post hoc power analysis based on the observed wound-healing difference also suggested high power in this subgroup, although this was considered supportive rather than primary because observed power is conditional on the detected effect size. By contrast, the smaller procedure-specific subgroups would be expected to have larger detectable thresholds and lower statistical precision and should therefore be interpreted as exploratory.

### Surgical procedures and patient characteristics

All procedures were performed by four senior orthopedic surgeons, each with over 10 years of experience. Each procedure type was consistently performed by the same lead surgeon to minimize inter-surgeon variability.

For rotator cuff repair, patients underwent arthroscopic repair using a standardized double-row suture bridge technique under general anesthesia with interscalene block. All cases used titanium suture anchors with nonabsorbable double-loaded sutures. Only full-thickness tears measuring 1–5 cm (DeOrio and Cofield classification) in the anterior–superior cuff were included. Tendon quality was assessed intraoperatively, and only cases with good reparability and tendon mobility were selected. Subacromial decompression and biceps tenotomy or tenodesis were performed as needed. Mean operative time was 91.5 ± 14.8 min. Preoperative MRI assessed tear size, tendon retraction, and fatty infiltration (Goutallier stages I–III).

In shoulder replacement, patients received cemented anatomic total shoulder arthroplasty via a deltopectoral approach. Integrity of the rotator cuff was supported intraoperatively, and cases requiring reverse TSA were excluded to maintain procedural homogeneity. The average operative time was 103.2 ± 19.1 min. Preoperative imaging classified glenohumeral arthritis severity (Samilson-Prieto) and glenoid morphology.

For subacromial decompression, all procedures were performed arthroscopically using standard posterior and lateral portals under general anesthesia. Subacromial bursectomy, coracoacromial ligament release, and acromioplasty were performed using a motorized burr. Only isolated impingement cases without rotator cuff pathology were included, supported by preoperative MRI and intraoperative findings. Mean operative time was 61.7 ± 10.3 min. All patients followed a standardized postoperative rehabilitation protocol.

### Control group method

In the CG, no PRP was administered. After completion of the surgical procedure, whether rotator cuff repair, shoulder replacement, or subacromial decompression, the surgical site was irrigated with sterile normal saline, hemostasis was achieved, and the wound was closed in layers using standard technique. A sterile pressure dressing was applied postoperatively.

### Treatment group method

Patients who received a single intraoperative PRP injection/application into the peri-incisional soft tissue immediately before skin closure.

### PRP preparation and characterization

PRP preparation and characterization Autologous platelet-rich plasma (PRP) was prepared intraoperatively under sterile conditions using an institutional standard protocol routinely applied at our tertiary referral center. Peripheral venous blood (30–60 mL) was collected into anticoagulant-containing tubes (ACD-A; blood:anticoagulant = 9:1, v/v) and processed within 30 min. PRP was produced using a two-step centrifugation method. Briefly, whole blood was first centrifuged at 250 × g for 10 min at room temperature to separate plasma from erythrocytes. The plasma layer was carefully aspirated while avoiding the buffy coat to minimize leukocyte carryover and transferred to a sterile tube. A second centrifugation was then performed at 800 × g for 10 min to concentrate platelets. After partial removal of the upper platelet-poor plasma, the platelet pellet was gently resuspended to yield a final PRP volume of approximately 4–6 mL per patient. PRP was used in liquid form without exogenous activation, as platelet activation was expected to occur physiologically upon contact with tissue collagen at the surgical site. Where laboratory documentation was available in the retrospective records, paired platelet and leukocyte counts in whole blood and final PRP were extracted for quality control. Given the retrospective nature of the study, laboratory characterization was not available for all patients. Based on the preparation method (buffy coat avoidance) and leukocyte profile, the product was classified as leukocyte-poor PRP (LP-PRP), consistent with commonly used PRP classification frameworks. Intraoperative application: Immediately before skin closure, PRP was delivered to the soft tissues surrounding the incision and the repair interface using a sterile syringe (target total volume approximately 5 mL, with minor adjustments according to the surgical field). No additional PRP was administered postoperatively. The same PRP preparation and application protocol was consistently applied across all surgery types, minimizing procedural variability between treatment groups.

### Postoperative treatment scheme

Rehabilitation details were retrospectively obtained from patient medical records. Standardized institutional protocols were used in routine care and were assumed to have been applied consistently across groups within each surgical subgroup.. Rotator cuff repair: Patients typically used an abduction brace for the first 3 weeks to protect the repair. During this period, wrist, elbow, and hand motion exercises were permitted. Passive range of motion of the shoulder was initiated from week 4, with gradual progression to active-assisted and then active movements by weeks 6–8. Resistance strengthening exercises were introduced approximately 12 weeks postoperatively.

Shoulder replacement: Patients were generally encouraged to begin passive and active-assisted shoulder motion within 2–3 weeks post-surgery, depending on pain tolerance and intraoperative cuff integrity. Bracing was used selectively. Strengthening and functional exercises typically commenced between weeks 6–8.

Subacromial decompression: Early mobilization was emphasized. Passive and active range of motion exercises were initiated during the first postoperative week. Strengthening exercises were introduced as tolerated beginning in weeks 4–6. Because of the retrospective design, rehabilitation adherence could not be quantitatively verified for all patients and was therefore not included as a covariate in the statistical models; this is acknowledged as a limitation.

### Main outcome measures

All procedures were performed by four senior orthopedic surgeons (each with > 10 years of experience). To reduce inter-surgeon variability, each procedure type was consistently led by the same surgeon.

For rotator cuff repair, arthroscopic repair was performed using a standardized double-row suture-bridge technique under general anesthesia with an interscalene block. Titanium suture anchors with nonabsorbable double-loaded sutures were used in all cases. Eligible tears were full-thickness lesions measuring 1–5 cm (DeOrio and Cofield classification) involving the anterosuperior cuff. Reparability was confirmed intraoperatively; only cases with adequate tendon mobility and acceptable tissue quality were included. Subacromial decompression and biceps tenotomy or tenodesis were performed when indicated. The mean operative time was 91.5 ± 14.8 min. Preoperative MRI was used to assess tear size, retraction, and fatty infiltration (Goutallier grades I–III).

For shoulder replacement, patients underwent cemented anatomic total shoulder arthroplasty via a deltopectoral approach. Rotator cuff integrity was confirmed intraoperatively, and cases requiring reverse total shoulder arthroplasty were excluded to maintain procedural consistency. The mean operative time was 103.2 ± 19.1 min. Preoperative imaging was used to grade glenohumeral osteoarthritis (Samilson–Prieto) and characterize glenoid morphology.

For subacromial decompression, all procedures were performed arthroscopically using standard posterior and lateral portals under general anesthesia. Subacromial bursectomy, coracoacromial ligament release, and acromioplasty were performed using a motorized burr. Only isolated impingement cases without rotator cuff pathology were included, based on preoperative MRI and intraoperative findings. The mean operative time was 61.7 ± 10.3 min. All patients followed a standardized postoperative rehabilitation protocol.

### Secondary observation index

Incidence rate of postoperative wound complications: Record all wound-related complications after operation, such as infection, wound dehiscence, hematoma, and scar hyperplasia. The incidence of complications was calculated, with the evaluation time set at 6 months post-operation.

Postoperative hospitalization time: Record the number of days required for patients from the end of surgery to discharge. The average hospitalization time of patients in TG and CG was compared to assess the effect of PRP therapy on postoperative recovery speed.

Patient satisfaction: Satisfaction data were obtained from routine follow-up records and standardized hospital feedback forms administered as part of clinical practice. Domains included pain control, mobility recovery, and overall care experience. Data were extracted retrospectively at the 6-month follow-up where available. No additional surveys were administered outside of standard care.

### Statistical method

All analyses were performed using SPSS version 22.0 (IBM Corp., Armonk, NY, USA). Continuous variables were summarized as mean ± standard deviation (SD) for approximately normally distributed data and as median (interquartile range, IQR) otherwise; categorical variables were presented as number (percentage). Normality was assessed using the Shapiro–Wilk test. In the unmatched cohort, baseline between-group comparisons were performed using independent-sample t tests or Mann–Whitney U tests, as appropriate, and categorical variables were compared using χ^2^ tests or Fisher’s exact tests.

Propensity score matching (PSM) was conducted separately within each surgical subgroup using 1:1 nearest-neighbor matching without replacement, with a caliper width of 0.2 SD of the logit of the propensity score. Covariate balance after matching was assessed using standardized mean differences (SMD), with SMD < 0.10 indicating adequate balance. Primary outcome analyses were performed in the matched cohort; post-matching between-group comparisons used paired-sample t tests or Wilcoxon signed-rank tests for continuous variables and McNemar’s test for categorical variables.

Repeated-measures outcomes (VAS and Constant scores across follow-up time points) were analyzed within each surgical subgroup using a mixed-design repeated-measures ANOVA, with treatment group (PRP vs non-PRP) as the between-subject factor and time as the within-subject factor; the group × time interaction was the primary effect of interest. Sphericity was assessed with Mauchly’s test, and the Greenhouse–Geisser correction was applied when sphericity was violated. Where post hoc pairwise comparisons were performed, Bonferroni adjustment was applied.

Because the primary cohort was created using 1:1 PSM, the repeated-measures ANOVA treated matched individuals as independent observations. To assess the potential impact of ignoring within-pair correlation, we performed a sensitivity analysis in the matched cohort using a paired-difference repeated-measures approach (calculating within-pair differences at each time point and testing time effects on these differences); results were consistent with the main analysis.

Repeated-measures analyses were conducted using complete cases across the specified time points (no imputation). The extent of missing data at each follow-up and the number of participants included in the repeated-measures analyses were reported. All tests were two-sided, and *P* < 0.05 was considered statistically significant unless otherwise specified.

## Result

### General baseline data

This study included 170 patients, consisting of 100 patients with rotator cuff repair (n = 100, 50 CG, 50 TG), 40 patients (20 CG, 20 TG) with shoulder replacement, and 30 patients (15 CG, 15 TG) with subacromial decompression. Within each surgical group, patients were matched between PRP and control groups based on key baseline characteristics, ensuring comparable surgical procedures. Post-matching, covariate balance was supported using standardized mean differences (< 0.1 for all variables), ensuring comparability.

Baseline variables, including age, sex, affected side, operation time, and preoperative platelet count, were statistically comparable between TG and CG in each surgical subgroup (*P* > 0.05 for all; see Table 1).

**Table 1:** Comparison of general baseline data.

Variable	Control group (n = 50)	Treatment group (n = 50)	*P*-value
Age (years)	57.1 ± 6.5	56.8 ± 6.2	0.73
Gender (male %)	38.0%	40.0%	0.78
Affected side (left %)	46.0%	44.0%	0.82
Operation time (min)	89.2 ± 20.3	90.1 ± 18.9	0.65
Platelet count (× 10^9/L)	235.2 ± 52.1	241.4 ± 50.7	0.54

Shoulder replacement	Control group (n = 20)	Treatment group (n = 20)	*P*-value
Age (years)	65.3 ± 7.1	64.7 ± 6.8	0.82
Gender (male %)	45.0%	50.0%	0.71
Affected side (left %)	40.0%	35.0%	0.66
Operation time (min)	103.1 ± 17.2	104.6 ± 18.5	0.74
Platelet count (× 10^9/L)	239.1 ± 49.8	242.7 ± 52.3	0.69

Subacromial decompression	Control group (n = 15)	Treatment group (n = 15)	*P*-value
Age (years)	51.8 ± 5.3	52.1 ± 5.1	0.89
Gender (male %)	35.0%	33.0%	0.80
Affected side (left %)	42.0%	45.0%	0.75
Operation time (min)	61.3 ± 9.8	62.2 ± 10.5	0.66
Platelet count (× 10^9/L)	231.4 ± 51.1	237.2 ± 48.7	0.58

### Follow-up and missing data

**Fig. 2:**
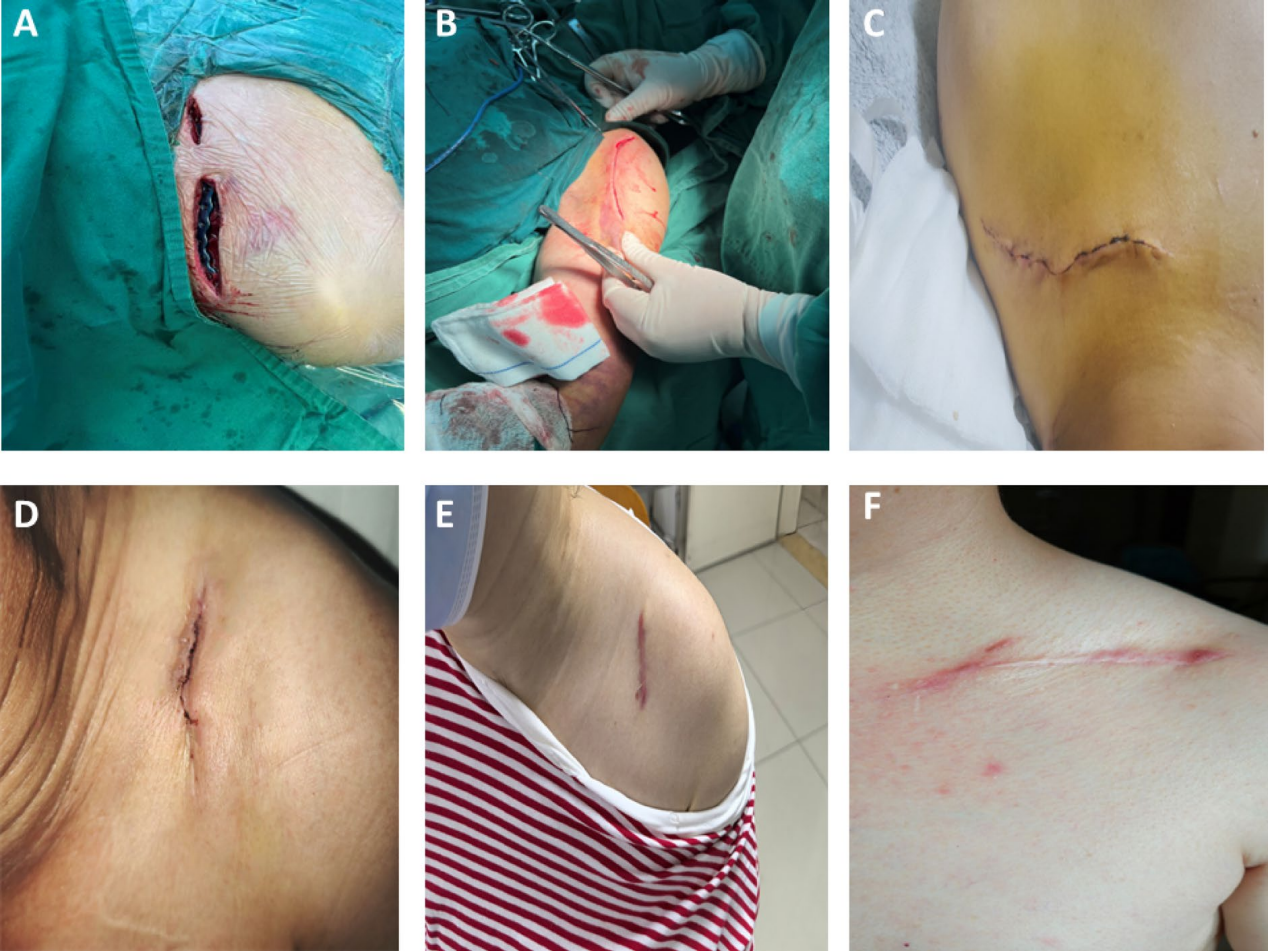
Representative images demonstrating the wound healing progression after shoulder surgery with intradermal suturing and PRP therapy. (**A**) Immediately before wound closure; (**B**) Immediately after intradermal suture placement; (**C**) 1-week post-surgery; (**D**) 2 weeks post-surgery, showing improved wound closure; (**E**) 3 months post-surgery, indicating healed incision with minimal scarring; (**F**) 6 months post-surgery, demonstrating mature scar formation and final wound appearance.

At 6 months, follow-up data were available for 82/85 (96.5%) in the PRP group and 81/85 (95.3%) in the control group. By procedure, 6-month follow-up was available for rotator cuff repair: 49/50 vs 48/50; shoulder replacement: 19/20 vs 20/20; subacromial decompression: 14/15 vs 13/15 (PRP vs control). Repeated-measures analyses were conducted using complete cases without imputation. Given the low attrition rate, missing data were considered unlikely to materially affect the main conclusions (Fig. 2).

### Comparison of wound healing time involving different surgeries types

**Fig. 3:**
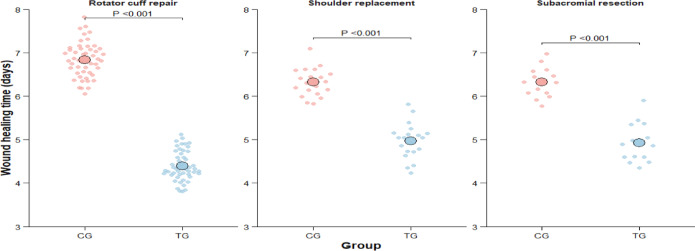
Wound healing time by surgery type and group. (**A**) Rotator cuff repair. (**B**) Shoulder replacement. (**C**) Subacromial decompression. Each dot represents an individual patient. The larger circles indicate group means. CG, control group; TG, treatment group. *P* values indicate between-group comparisons within each surgical subgroup (two-tailed; significance threshold as defined in the Methods).

Wound-healing duration differed by surgical procedure, but was consistently shorter in the PRP group across all three surgical subgroups in the propensity score–matched cohort (Table 2; Fig. 3). After rotator cuff repair, mean healing time was 4.45 ± 0.31 days in the PRP group versus 6.78 ± 0.42 days in the non-PRP group, corresponding to a mean difference (PRP − non-PRP) of − 2.33 days (95% CI, − 2.51 to − 2.15; *P* < 0.001). Similar findings were observed in shoulder replacement (4.92 ± 0.38 vs 6.35 ± 0.41 days; mean difference, − 1.43 days; 95% CI, − 1.67 to − 1.19; *P* < 0.001) and subacromial decompression (4.67 ± 0.35 vs 6.23 ± 0.39 days; mean difference, − 1.56 days; 95% CI, − 1.85 to − 1.27; *P* < 0.001). To assess heterogeneity across procedures, an interaction term between surgery type and treatment group was evaluated, demonstrating a significant surgery type × group interaction (*P*_interaction = 0.003), indicating that the magnitude of the PRP-associated reduction in healing time varied by procedure, with the largest absolute reduction observed after rotator cuff repair. Representative wound-progression photographs are shown in Fig. 2.

**Table 2:** Subgroup analysis of wound healing time by surgery type.

Surgery Type	CG Mean ± SD (days)	TG Mean ± SD (days)	Mean Difference (95% CI)	*P*-value
Rotator cuff repair (n = 50 each group)	6.78 ± 0.42	4.45 ± 0.31	− 2.33 (− 2.51 to − 2.15)	< 0.001*
Shoulder replacement (n = 20 each group)	6.35 ± 0.41	4.92 ± 0.38	− 1.43 (− 1.67 to − 1.19)	< 0.001*
Subacromial decompression (n = 15 each group)	6.23 ± 0.39	4.67 ± 0.35	− 1.56 (− 1.85 to − 1.27)	< 0.001*

(*P < 0.01).

### Comparison of VAS pain score between two groups

**Fig. 4:**
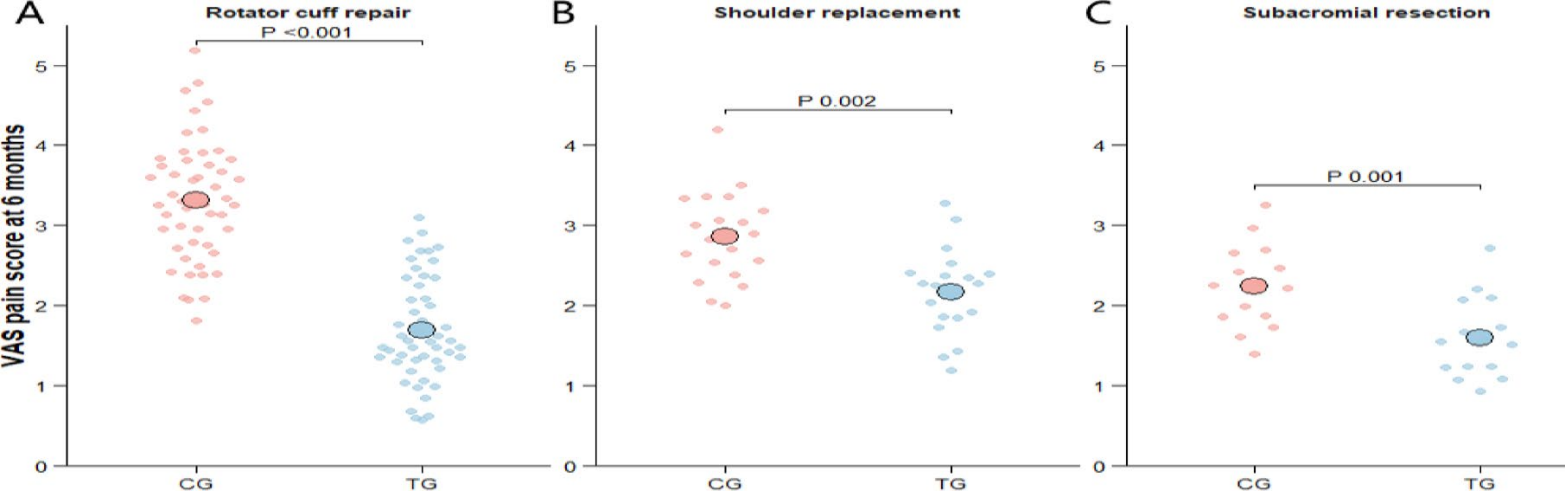
Comparison of VAS pain scores at 6 months between groups. (**A**) Rotator cuff repair: the treatment group (TG) showed significantly lower VAS pain scores at 6 months compared with the control group (CG) (*P* < 0.001). (**B**) Shoulder replacement: VAS pain scores at 6 months were significantly lower in the TG than in the CG (*P* = 0.002). (**C**) Subacromial decompression: the TG showed significantly is associated with reduced VAS pain scores at 6 months compared with the CG (*P* = 0.001). Note: Each dot represents an individual patient. Larger circles indicate group means. CG, control group; TG, treatment group. VAS, visual analog scale (range 0–10), with higher scores indicating greater pain intensity. Statistical significance is indicated by the corresponding *P* values.

At the 6-month follow-up, patients in the PRP group reported significantly lower VAS pain scores than those in the control group across all surgical subgroups (Table 3, Fig. 4). In the rotator cuff repair subgroup, the mean VAS score was 1.8 ± 0.6 in the PRP group versus 3.2 ± 0.8 in the control group (mean difference, − 1.4; 95% CI, − 1.7 to − 1.1; *P* < 0.001). Among shoulder replacement patients, VAS scores were also lower in the PRP group (2.1 ± 0.5 vs. 2.9 ± 0.7; mean difference, − 0.8; 95% CI, − 1.2 to − 0.4; *P* = 0.002). Subacromial decompression patients showed a similar pattern (1.3 ± 0.4 vs. 2.1 ± 0.6; mean difference, − 0.8; 95% CI, − 1.2 to − 0.4; *P* = 0.001). Effect modification analysis did not identify a statistically significant interaction between surgery type and treatment group for 6-month VAS scores, suggesting no evidence of differential PRP-associated pain reduction across surgical procedures (surgery type × group interaction, *P* = 0.21).

**Table 3:** analysis of vas pain scores by surgery type at 6 months post-operation.

Surgery Type	CG (Mean ± SD)	TG (Mean ± SD)	Mean Difference (95% CI)	*P*-value
Rotator cuff repair (n = 50 each group)	3.2 ± 0.8	1.8 ± 0.6	− 1.4 (− 1.7 to − 1.1)	< 0.001*
Shoulder replacement (n = 20 each group)	2.9 ± 0.7	2.1 ± 0.5	− 0.8 (− 1.2 to − 0.4)	0.002*
Subacromial decompression (n = 15 each group)	2.1 ± 0.6	1.3 ± 0.4	− 0.8 (− 1.2 to − 0.4)	0.001*

Values are presented as mean ± SD at 6 months post-operation. Mean difference is TG − CG (95% CI). *P*-values represent between-group comparisons within each surgery type. No evidence of effect modification by procedure was observed (surgery type × group interaction, *P* = 0.21). *P < 0.01. Numbers reflect complete cases with available 6-month VAS data.

### Comparison of constant score between the two groups

**Fig. 5:**
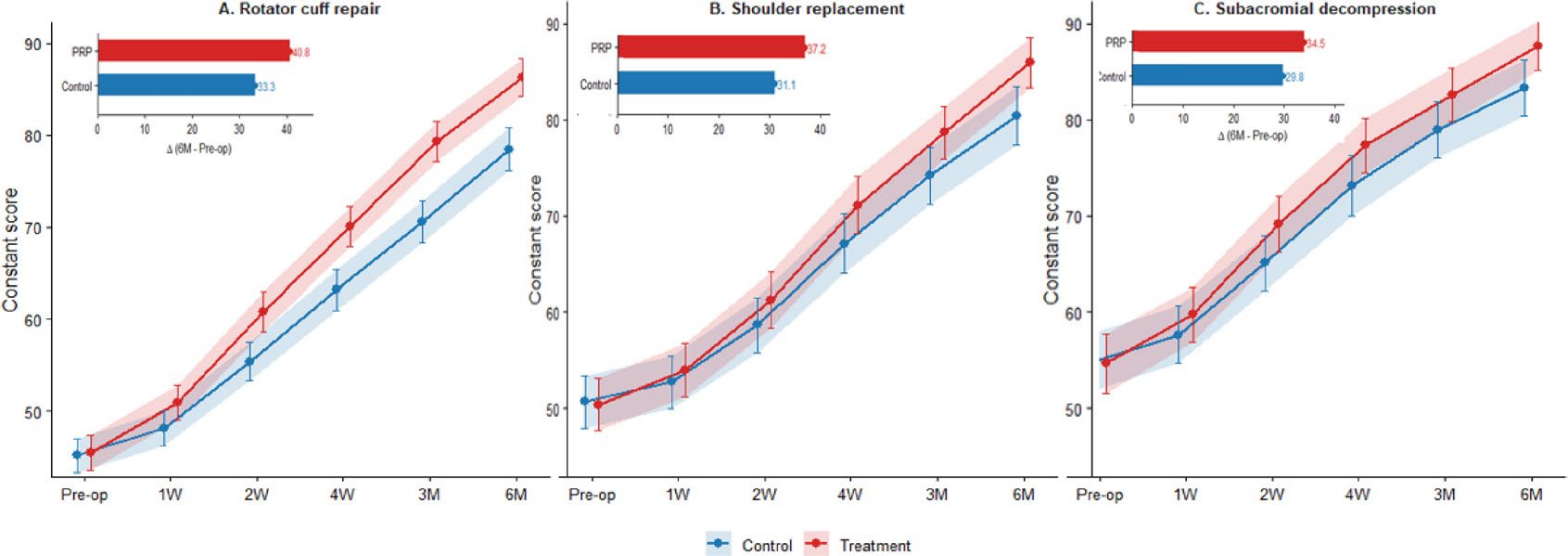
Constant score trajectories over time by surgery type. (**A**) Rotator cuff repair. (**B**) Shoulder replacement. (**C**) Subacromial decompression. Mean Constant scores are shown at each follow-up time point (pre-operation, 1 week, 2 weeks, 4 weeks, 3 months, and 6 months) for the Control group (blue) and Treatment/PRP group (red). Shaded bands represent 95% confidence intervals. Error bars indicate uncertainty around the mean at each time point. Insets summarize the change from baseline to 6 months [Δ(6 M–Pre-op)] for each group within the corresponding surgical subgroup. Shaded bands represent 95% CIs; vertical error bars represent SEM.

**Fig. 6:**
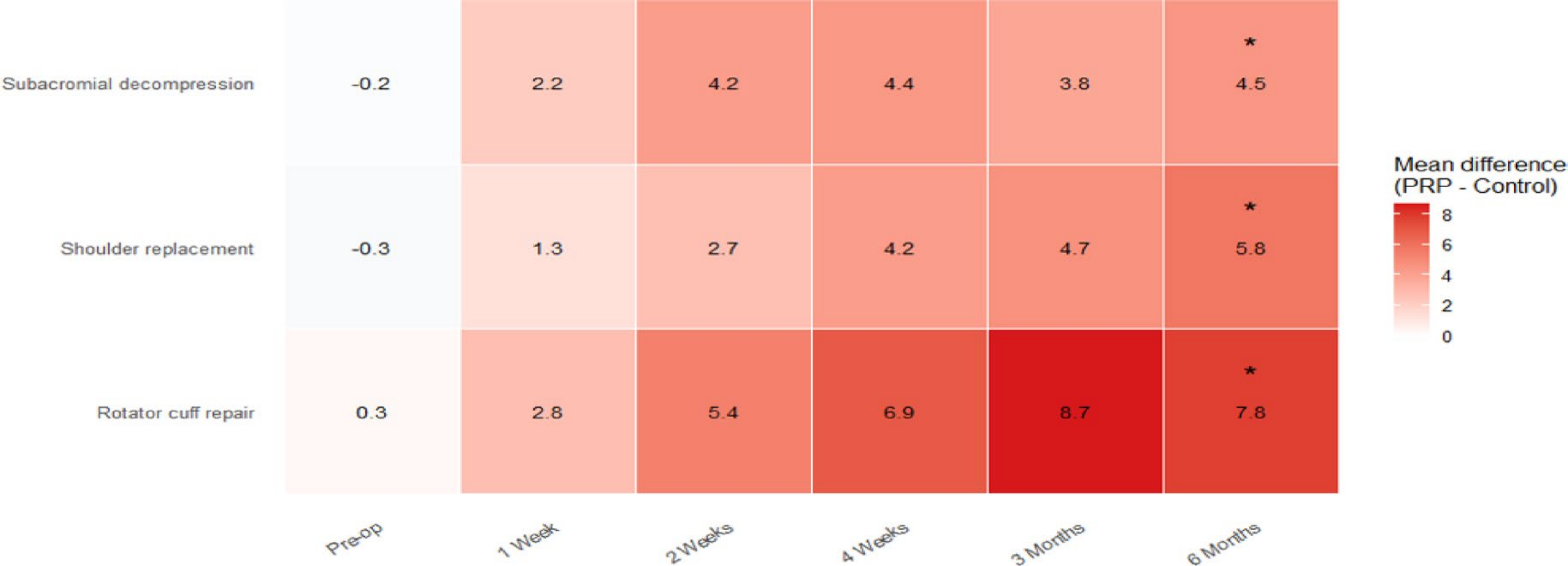
PRP–control differences in Constant score across follow-up and surgery type. Heatmap cells display the mean between-group difference in Constant score (PRP minus control, points) at each time point (pre-op, 1 week, 2 weeks, 4 weeks, 3 months, and 6 months) within each surgical subgroup: rotator cuff repair (n = 50 per group), shoulder replacement (n = 20 per group), and subacromial decompression (n = 15 per group). Values printed in each cell correspond to the mean differences; darker shading indicates a larger advantage for PRP. Asterisks denote 6-month comparisons that met the prespecified multiple-comparison criterion (Bonferroni-adjusted alpha = 0.01).

Functional recovery patterns varied among surgical subgroups (Table 4, Figs. 5, 6). In the rotator cuff repair cohort (n = 100, 50 CG, 50 TG), Constant scores were higher in the TG group at 2 weeks (60.8 ± 7.5 vs. 55.4 ± 7.3), 4 weeks (70.1 ± 7.6 vs. 63.2 ± 7.9), 3 months (79.3 ± 7.5 vs. 70.6 ± 8.1), and 6 months (86.3 ± 7.1 vs. 78.5 ± 8.2), with between-group differences meeting the prespecified multiple-comparison criterion at 6 months (*P* < 0.001; Table 4) and consistent PRP advantages across intermediate time points (Fig. 5A). Shoulder replacement patients (n = 40, 20 CG, 20 TG) showed a sustained separation in Constant scores from 4 weeks onward, reaching 87.9 ± 5.9 in the TG group versus 82.1 ± 6.8 in controls at 6 months (*P* = 0.003; Table 4; Fig. 5B). In the subacromial decompression cohort (n = 30, 15 CG, 15 TG), Constant scores also favored TG at later follow-up, with 6-month scores of 89.7 ± 4.8 versus 85.2 ± 5.5 (*P* = 0.009; Table 4; Fig. 5C). The heatmap of PRP–control differences (Fig. 6) showed progressive between-group separation from 1 week onward, with the largest mean difference observed after rotator cuff repair at 3 months (8.7 points) and sustained differences at 6 months across all subgroups (7.8, 5.8, and 4.5 points for rotator cuff repair, shoulder replacement, and subacromial decompression, respectively). Across the three procedures, a formal surgery type × group interaction test for the 6-month PRP–control mean difference was not significant (*P* for interaction = 0.38), supporting a broadly consistent direction of PRP-associated functional improvement across surgical subgroups (Table 4, Fig. 6).

**Table 4:** Subgroup analysis of constant scores by surgery type.

Surgery type	Group	Pre-op	1 Week	2 Weeks	4 Weeks	3 Months	6 Months	*P*-value (6 M)
Rotator cuff repair	Control	45.2 ± 6.5	48.1 ± 6.7	55.4 ± 7.3	63.2 ± 7.9	70.6 ± 8.1	78.5 ± 8.2	< 0.001
Rotator cuff repair	PRP	45.5 ± 6.8	50.9 ± 6.6	60.8 ± 7.5	70.1 ± 7.6	79.3 ± 7.5	86.3 ± 7.1	
Shoulder replacement	Control	51.0 ± 6.0	53.2 ± 6.1	59.4 ± 6.4	68.2 ± 6.9	75.6 ± 6.7	82.1 ± 6.8	0.003
Shoulder replacement	PRP	50.7 ± 6.1	54.5 ± 6.2	62.1 ± 6.5	72.4 ± 6.6	80.3 ± 6.1	87.9 ± 5.9	
Subacromial decompression	Control	55.4 ± 5.7	58.3 ± 5.6	66.1 ± 5.4	74.5 ± 5.9	80.6 ± 5.6	85.2 ± 5.5	0.009
Subacromial decompression	PRP	55.2 ± 5.8	60.5 ± 5.4	70.3 ± 5.5	78.9 ± 5.3	84.4 ± 5.2	89.7 ± 4.8	

Data presented as mean ± SD. *P*-values are adjusted for multiple comparisons using Bonferroni correction (*P < 0.01). Numbers reflect available cases at each time point; 6-month availability is summarized in Supplementary Table S1.

### Sensitivity analysis (linear mixed-effects model)

A sensitivity analysis using a linear mixed-effects model (LMM), which incorporates all available observations, showed results consistent with the primary analyses. The estimated PRP–control differences increased over time and were largest at 6 months across subgroups (approximately + 7.8 points for rotator cuff repair, + 5.8 points for shoulder replacement, and + 4.5 points for subacromial decompression). Approximate LMM contrasts suggested statistically significant differences at 6 months in all three subgroups (Supplementary Table S1 Supplementary Table S2).

### Comparison of complications in different surgery type groups

Complications analysis revealed surgery-specific patterns (Table 5), graded according to the National Cancer Institute Common Terminology Criteria for Adverse Events (NCI-CTCAE). In the rotator cuff repair subgroup, the PRP group had a numerically lower complication rate than the control group (2.0% vs. 8.0%); however, this difference was not statistically significant (Fisher’s exact test, *P* = 0.362; RR = 0.25, 95% CI 0.03–2.15). In the shoulder replacement and subacromial decompression subgroups, complication rates were identical between groups (5.0% and 6.7%, respectively), with no significant differences (*P* = 1.000 for both).

**Table 5:** Complication rates by shoulder surgery type.

Surgery type	CG	TG	*P*-value	Relative risk (TG/CG, 95% CI)
Rotator cuff repair (n = 50 each group)	4/50 (8.00%)	1/50 (2.00%)	0.362	0.25 (0.03 to 2.15)
Shoulder replacement (n = 20 each group)	1/20 (5.00%)	1/20 (5.00%)	1.000	1.00 (0.07 to 14.90)
Subacromial decompression (n = 15 each group)	1/15 (6.67%)	1/15 (6.67%)	1.000	1.00 (0.07 to 14.55)

### Comparison of hospitalization time involving the two groupings

Hospitalization duration differed across procedures (Table 6). In rotator cuff repair, the PRP group had a shorter length of stay than controls (6.12 ± 0.88 vs 11.25 ± 1.35 days; *P* < 0.001). Similar between-group differences were observed in shoulder replacement (7.15 ± 1.12 vs 10.85 ± 1.58 days; *P* < 0.001) and subacromial decompression (6.23 ± 0.95 vs 9.17 ± 1.24 days; *P* < 0.001). Overall, PRP use was associated with shorter hospitalization across these shoulder procedures.

**Table 6:** Subgroup analysis of hospitalization time by surgery type.

Surgery Type	CG	TG	Mean difference (95% CI)	*P*-value
Rotator cuff repair (n = 50 each group)	11.25 ± 1.35	6.12 ± 0.88	− 5.13 (− 5.62 to − 4.64)	< 0.001*
Shoulder replacement (n = 20 each)	10.85 ± 1.58	7.15 ± 1.12	− 3.70 (− 4.52 to − 2.88)	< 0.001*
Subacromial decompression (n = 15 each group)	9.17 ± 1.24	6.23 ± 0.95	− 2.94 (− 3.62 to − 2.26)	< 0.001*

(*P < 0.01).

### Comparison of patients’ satisfaction between two groups

Table 7 shows patient satisfaction by group and procedure. Overall satisfaction (very satisfied or satisfied) was higher in the treatment group than in the control group (78/85, 91·8% vs 64/85, 75·3%; χ^2^ = 6·127; *P* = 0·013).

**Table 7:** Comparative analysis of patient satisfaction involving the two groupings.

		Very Satisfied n(%)	Satisfied n(%)	Common n(%)	Dissatisfied n(%)	Total satisfaction rate	χ^2^	*P*
Rotator cuff repair	Control (50)	19 (38.0%)	19 (38.0%)	4 (8.0%)	8 (16.0%)	38/50 = 76.0%	4.762	0.029
	Treatment (50)	23 (46.0%)	23 (46.0%)	4 (8.0%)	0 (0.0%)	46/50 = 92.0%		
Shoulder replacement	Control (20)	10 (50.0%)	5 (25.0%)	2 (10.0%)	3 (15.0%)	15/20 = 75.0%	1.558	0.212
	Treatment (20)	4 (20.0%)	14 (70.0%)	0 (0.0%)	2 (10.0%)	18/20 = 90.0%		
Subacromial decompression	Control (15)	2 (13.3%)	9 (60.0%)	4 (26.7%)	0 (0.0%)	11/15 = 73.3%	2.160	0.142
	Treatment (15)	11 (73.3%)	3 (20.0%)	0 (0.0%)	1 (6.7%)	14/15 = 93.3%		
All	Control group (n = 85)	31 (36.5%)	33 (38.8%)	10 (11.8%)	11 (12.9%)	64/85 = 75.3%	6.127	0.013
	Treatment group (n = 85)	38 (44.7%)	40 (47.1%)	4 (4.7%)	3 (3.5%)	78/85 = 91.8%		

Data are presented as n (%). “Total satisfaction rate” was defined a priori as the proportion of patients reporting “very satisfied” or “satisfied”. Comparisons between the PRP and control groups within each procedure were performed using the χ^2^ test (Fisher’s exact test was applied when expected counts were < 5). Given the limited sample sizes in the shoulder replacement and subacromial decompression subgroups, these subgroup analyses are descriptive and should be interpreted as hypothesis-generating rather than confirmatory. A two-sided *P* value < 0.05 was considered statistically significant.

For rotator cuff repair, satisfaction was 46/50 (92·0%) in the treatment group and 38/50 (76·0%) in the control group (χ^2^ = 4·762; *P* = 0·029). For shoulder replacement, satisfaction was 18/20 (90·0%) versus 15/20 (75·0%), respectively (χ^2^ = 1·558; *P* = 0·212). For subacromial decompression, satisfaction was 14/15 (93·3%) in the treatment group and 11/15 (73·3%) in the control group (χ^2^ = 2·160; *P* = 0·142).

## Discussion

Due to its complex structure and frequent use, the shoulder is often susceptible to various sports injuries and degenerative diseases. Shoulder surgery is commonly performed to treat a range of shoulder ailments and injuries. Common indications for shoulder surgery include rotator cuff tears, shoulder inflammation, subacromial impingement syndrome, and shoulder instability^17^. Through shoulder surgery, pain can be effectively relieved by repairing torn tendons, removing inflammatory tissue, repairing joint surfaces, or performing joint replacement surgery, thereby improving joint function^18–20^. After shoulder surgery, patients need to go through a certain rehabilitation process, and there are also some challenges in wound healing and functional recovery^21^. In this context, the introduction of PRP technology offers a potential adjunctive treatment option. PRP can promote tissue repair and regeneration, accelerate wound healing, and has been reported to be associated with reduced postoperative complications, and may support surgical outcomes by concentrating patients’ own platelets and injecting them into the surgical site^22^.

Propensity score matching was used to create a 1:1 matched control group with similar baseline clinical characteristics, allowing a more balanced assessment of the association between PRP therapy and postoperative outcomes after shoulder surgery. PRP therapy was associated with a shorter time to documented wound healing, a finding that is biologically plausible given the known effects of PRP on tissue repair. Skin-incision healing time after shoulder surgery has received limited attention in the literature, and these results therefore provide real-world evidence for an understudied outcome. They further suggest that intraoperative local application of PRP to peri-incisional soft tissue may be associated with faster wound healing, although prospective confirmation is needed.

The improvement in shoulder function, reflected by higher Constant-Murley scores, may partly relate to reduced pain and improved tissue repair, both of which may support more effective rehabilitation. Although PRP has been reported to improve functional outcomes after rotator cuff repair, possibly through effects on tendon healing, the evidence remains much more limited for other shoulder procedures^23^. Similarly, Castricini et al. reported improved functional scores with PRP in rotator cuff repairs, yet called for broader studies across surgical types^24^. This gap in the literature highlights the need to evaluate the functional effects of PRP across different shoulder procedures. In the present study, subgroup analyses suggested that PRP may be associated with improved functional recovery after rotator cuff repair, shoulder replacement, and subacromial decompression. These findings should be interpreted cautiously, however, as the subgroup analyses were exploratory and some procedure groups were relatively small. It is therefore possible that any benefit of PRP is more apparent in procedures involving greater soft-tissue injury, but this will require confirmation in adequately powered prospective studies^25^. By extending PRP’s functional benefits beyond rotator cuff repairs, our study provides a more comprehensive understanding of its role in shoulder surgery, informing clinical decision-making for procedure-specific applications. In the rotator cuff repair cohort, the between-group difference in Constant score at 6 months was statistically significant, but may be below commonly cited MCID thresholds reported in recent literature, and thus the average clinical magnitude should be interpreted cautiously^26^.

PRP’s anti-inflammatory properties are hypothesized to mitigate postoperative pain by reducing the inflammatory response at the surgical site. A randomized controlled trial reported is associated with reduced pain scores in rotator cuff repair patients treated with PRP, particularly in the early postoperative period^27^. However, a meta-analysis in Arthroscopy found inconsistent pain relief across studies, attributing variability to differences in PRP preparation and surgical techniques^28^. These studies primarily focused on rotator cuff repairs, leaving a gap in understanding PRP’s pain-relieving effects across other shoulder surgeries, such as shoulder replacement or arthroscopy. Our subgroup analyses address this gap by suggesting that PRP was associated with lower pain scores in more invasive procedures, including rotator cuff repair and shoulder replacement, where inflammatory burden is greater, while minimal or no significant effects were observed in arthroscopic procedures. These findings suggest that PRP’s potential anti-inflammatory benefits may be more pronounced in surgeries involving greater tissue disruption, which could enhance patient comfort and facilitate earlier rehabilitation^29^. By clarifying these procedure-specific trends, our study may help inform more targeted use of PRP in postoperative pain management, ensuring that it is applied where it is most likely to offer clinical benefi**t**^30^.

PRP’s impact on secondary outcomes, including complication rates, hospital stay duration, and patient satisfaction, further underscores its clinical utility. A previous study reported that PRP was associated with reduced complication rates in orthopedic procedures, but data on hospital stay and patient satisfaction remain sparse^31^.A previous study noted PRP’s potential association with reduced complications in tendon injuries , yet did not explore broader surgical contexts^32^. Our study helps address this gap by showing that PRP use was associated with fewer postoperative complications, with the most consistent signal observed in rotator cuff repair, and with a shorter length of hospital stay across procedures. These observations may suggest faster early recovery; however, while a similar trend toward reduced hospitalization was seen across surgery types, the findings in the shoulder replacement and subacromial decompression subgroups should be interpreted as hypothesis-generating because of limited sample size and potential residual confounding. Moreover, length of stay is influenced by both clinical and non-clinical factors (e.g., local discharge policies, rehabilitation availability, and social support), and therefore the observed reduction in hospitalization should be interpreted cautiously and ideally confirmed alongside standardized discharge criteria in prospective studies. Higher patient satisfaction, likely driven by improved pain control and functional recovery, highlights PRP’s role in enhancing the postoperative experience^33,34^. These findings suggest that PRP not only supports biological outcomes but also contributes to healthcare efficiency by reducing hospital stays, particularly in invasive surgeries^35^. From a health-economic standpoint, earlier discharge may translate into lower inpatient resource use and reduced overall cost, although formal cost-effectiveness analyses were beyond the scope of this retrospective study and should be examined prospectively. The limited benefit seen in the arthroscopy cohort also suggests that the clinical value of PRP may be procedure-specific, with implications for cost-effectiveness in routine practice. Importantly, PRP in the present study was applied locally at wound closure. This differs from reports describing PRP injection months before total shoulder arthroplasty, where a potential increase in infection risk has been discussed, suggesting that timing and route of administration may both be clinically relevant^36^.

There are several limitations to this study. First, it was conducted at a single center and used a retrospective design. Although propensity score matching improved observed baseline comparability, it cannot account for unmeasured confounding and does not substitute for randomization; therefore, residual confounding and some degree of selection bias cannot be excluded, which may limit generalizability. Second, the control group did not receive a placebo or sham injection, and blinded assessment was not feasible in the original clinical setting. This may have introduced bias, particularly for subjective outcomes such as pain and functional scores. Third, PRP preparation was not fully standardized or biologically characterized across patients, including platelet concentration, leukocyte content, and activation protocol, which may have contributed to heterogeneity in treatment response and may limit reproducibility. Fourth, adherence to rehabilitation could not be reliably captured from routine records and was therefore not included in the analysis, although it may have influenced functional recovery. In addition, wound-healing time was derived from retrospective clinical documentation and reflected the first recorded confirmation of healing criteria rather than the exact biological time of wound closure; variation in documentation, especially after discharge, may therefore have introduced measurement imprecision. The subgroup analyses by surgery type were based on relatively small numbers and should be regarded as exploratory; accordingly, any apparent procedure-specific effects should be interpreted as hypothesis-generating rather than definitive. Length of hospital stay should likewise be interpreted cautiously, as it may have been influenced by non-clinical factors such as discharge practice, rehabilitation availability, and social support. Larger multicenter prospective studies with standardized PRP protocols, predefined wound-assessment methods, and surgery-specific randomization will be required to confirm these findings and determine whether the apparent procedure-specific differences are reproducible.

## Conclusion

PRP therapy was associated with faster wound healing, less postoperative pain, and better shoulder function, with the clearest benefits seen after more invasive procedures such as rotator cuff repair and shoulder replacement. These findings suggest that PRP was associated with procedure-specific differences in postoperative recovery, potentially through improved pain control and facilitation of earlier rehabilitation. Although this study provides real-world evidence across different shoulder surgeries, PRP preparation methods and clinical indications require further standardization, and prospective multicenter studies are needed before firm clinical recommendations can be made. Larger multicenter prospective studies with longer follow-up are needed to confirm generalizability and long-term effectiveness.

## Supplementary Information

Below is the link to the electronic supplementary material.

Supplementary Material 1

## Data Availability

The datasets used and/or analyzed during the current study are available from the corresponding author on reasonable request.
