# Supplementary material for: Effectiveness of platelet-rich plasma therapy in promoting wound healing and shoulder function recovery after shoulder surgery
Source: Sci Rep. 2026 Mar 27;16:10894. doi: 10.1038/s41598-026-44844-3 (PMC13039741; doi:10.1038/s41598-026-44844-3)
Supplement: Supplementary file 1 — Supplementary Material 1 [file 41598_2026_44844_MOESM1_ESM.docx]

**Supplementary Table S1 Availability of Constant score data at 6 months**

| Procedure | PRP available / total (%) | Control available / total (%) |
| --- | --- | --- |
| Rotator cuff repair | 49/50 (98.0%) | 48/50 (96.0%) |
| Shoulder replacement | 19/20 (95.0%) | 20/20 (100%) |
| Subacromial decompression | 14/15 (93.3%) | 13/15 (86.7%) |
| Overall | 82/85 (96.5%) | 81/85 (95.3%) |

**Supplementary Table S2. LMM sensitivity analysis**

| Surgery type | Time point | PRP–Control (points) | 95% CI | P |
| --- | --- | --- | --- | --- |
| Rotator cuff repair | 1 week | +2.8 | +0.2 to +5.4 | 0.035 |
|  | 2 weeks | +5.4 | +2.5 to +8.3 | <0.001 |
|  | 4 weeks | +6.9 | +3.9 to +9.9 | <0.001 |
|  | 3 months | +8.7 | +5.6 to +11.8 | <0.001 |
|  | 6 months | +7.8 | +4.7 to +10.9 | <0.001 |
| Shoulder replacement | 1 week | +1.3 | −2.5 to +5.1 | 0.504 |
|  | 2 weeks | +2.7 | −1.3 to +6.7 | 0.186 |
|  | 4 weeks | +4.2 | +0.0 to +8.4 | 0.049 |
|  | 3 months | +4.7 | +0.7 to +8.7 | 0.020 |
|  | 6 months | +5.8 | +1.8 to +9.8 | 0.004 |
| Subacromial decompression | 1 week | +2.2 | −1.7 to +6.1 | 0.273 |
|  | 2 weeks | +4.2 | +0.3 to +8.1 | 0.035 |
|  | 4 weeks | +4.4 | +0.4 to +8.4 | 0.032 |
|  | 3 months | +3.8 | −0.1 to +7.7 | 0.054 |
|  | 6 months | +4.5 | +0.6 to +8.4 | 0.024 |

Sensitivity analysis using a linear mixed-effects model (LMM). Differences represent estimated PRP–Control contrasts at each post-operative time point within each surgery type. Values shown here are approximate, derived from group means and standard deviations with assumed small attrition, and are provided for internal drafting purposes only
